# Left ventricular myocardial strain and tissue characterization by cardiac magnetic resonance imaging in immune checkpoint inhibitor associated cardiotoxicity

**DOI:** 10.1371/journal.pone.0246764

**Published:** 2021-02-19

**Authors:** Angela Y. Higgins, Amit Arbune, Aaron Soufer, Elio Ragheb, Jennifer M. Kwan, Jerome Lamy, Mariana Henry, Jason R. Cuomo, Ahmad Charifa, Cesia Gallegos, Sarah Hull, Jessica Shank Coviello, Anna S. Bader, Dana C. Peters, Steffen Huber, Hamid R. Mojibian, Albert J. Sinusas, Harriet Kluger, Lauren A. Baldassarre

**Affiliations:** 1 Department of Internal Medicine, Section of Cardiovascular Medicine, Yale University School of Medicine, New Haven, Connecticut, United States of America; 2 Department of Radiology and Biomedical Imaging, Yale School of Medicine, New Haven, Connecticut, United States of America; 3 Department of Pathology, Oregon Health & Science University, Portland, Oregon, United States of America; 4 Yale School of Nursing and Smilow Cancer Hospital, New Haven, Connecticut, United States of America; 5 Department of Biomedical Engineering, Yale University School of Engineering and Applied Science, New Haven, CT, United States of America; 6 Department of Internal Medicine, Section of Medical Oncology, Yale University School of Medicine, New Haven, Connecticut, United States of America; Faculty of Medical Science - State University of Campinas, BRAZIL

## Abstract

**Background:**

Immune checkpoint inhibitors (ICIs) are highly effective in treating cancer; however, cardiotoxicity can occur, including myocarditis. Cardiac magnetic resonance (CMR) imaging is useful for evaluation of myocarditis, although it has not been well studied in ICI cardiotoxicity.

**Methods:**

We identified patients referred for CMR evaluation of ICI cardiotoxicity from September 2015 through September 2019. We assessed structural and functional parameters, feature tracking (FT) left ventricular and atrial strain, T2- weighted ratios and quantitative late gadolinium enhancement (LGE). We also applied the Updated Lake Louise Criteria for diagnosis of myocarditis.

**Results:**

Of the 20 patients referred, the median left ventricular ejection fraction (LVEF) was 52.5% ± 19.1 and 50% had a normal LVEF (≥53%). FT strain analysis revealed an average abnormal global longitudinal strain (GLS) of −9.8%± 4.2%. In patients with a normal LVEF, the average GLS remained depressed at −12.3%± 2.4%. In all patients, GLS demonstrated a significant negative correlation with LVEF (r_s_ = −0.64, p 0.002). Sixteen patients (80%) had presence of LGE (14 non-ischemic pattern and 2 ischemic). Percent LGE did not correlate with any CMR parameters and notably did not correlate with LVEF (r_s_ = −0.29, p = 0.22) or GLS (r_s_ = 0.10, p = 0.67), highlighting the value of tissue characterization beyond functional assessment. Nine patients (45%) met full Updated Lake Louise Criteria and 85% met at least one criterion, suggestive of myocarditis in the correct clinical context. Thirteen patients (65%) were treated for ICI-associated myocarditis and, of these, 54% (n = 7) had recovery of LVEF to normal. There was no correlation between LVEF (p = 0.47), GLS (0.89), or % LGE (0.15) and recovery of LVEF with treatment.

**Conclusion:**

In patients with suspected ICI cardiotoxicity, CMR is an important diagnostic tool, even in the absence of overt left ventricular dysfunction, as abnormalities in left ventricular strain, T2 signal and LGE can identifying disease.

## Introduction

Recent advances in immunotherapy have improved survival in cancer patients. The two FDA-approved classes of immune checkpoint inhibitors (ICIs) inhibit T-cell activation by targeting cytotoxic T lymphocyte antigen 4, programmed cell death protein-1 or programmed cell death ligand 1. ICIs have revolutionized the field of immuno-oncology, as many patients have seen immediate and lasting beneficial effects, and the number of trials utilizing ICIs is growing rapidly [1]. The use of ICIs is limited by immune-related adverse events, including myocarditis [2]. Cardiotoxicity was rare in clinical trials; however, cardiac surveillance was limited with no prospective assessment of cardiac function. There have now been many case publications, cases series and a retrospective study of ICI cardiotoxicity, with a reported prevalence of up to 1.1% [3, 4]. Recent meta-analysis found that 9.8% of ICI treatment-related deaths were cardiovascular [5]. Myocarditis is the most frequently reported cardiotoxicity; however, pericarditis, heart failure with reduced LVEF, arrhythmia, myocardial infarction and cardiac arrest have also been reported as initial presentations [4, 6]. The incidence of major adverse cardiac events (MACE) in patients with ICI associated myocarditis has been reported as almost 50% [3].

Currently there is no standard for the diagnosis and management of patients with ICI cardiotoxicity. Cardiac magnetic resonance (CMR) imaging is a well-established tool for diagnosis of myocarditis in general and provides detailed tissue characterization, in addition to functional data, which can increase the sensitivity for detection of myocarditis [7, 8]. Myocardial edema assessment with T2-weighted (T2W) imaging and myocardial fibrosis imaging with late gadolinium enhancement (LGE) are the two primary techniques employed for CMR evaluation of myocarditis and are incorporated into the well accepted Lake Louise Criteria [7, 8]. A recent retrospective study reported a relatively low incidence of LGE and abnormal T2W signal in patients with ICI myocarditis [9]. However, this was a real-world study which utilized qualitative assessment of LGE and T2W signal from clinical reports at multiple different sites, highlighting the need for more quantitative and systematic assessment for increased sensitivity.

Myocardial strain by echocardiography and CMR has been extensively studied in chemotherapy associated cardiotoxicity [10–12]. Additionally, abnormal myocardial strain by CMR has been reported in patients with non-ICI suspected myocarditis with normal LVEF [13, 14]. More recently, a reduction in global longitudinal strain (GLS) by echocardiography has been seen with ICI associated myocarditis, independent of LVEF, and was associated with MACE [15]. Left atrial strain has not been evaluated in ICI cardiotoxicity but has been shown to have incremental value in other types of myocarditis [16]. Similarly, strain by CMR has not been assessed specifically in ICI associated myocarditis.

CMR is well established in the diagnosis of myocarditis; however, the recent study using only qualitative CMR data in ICI myocarditis suggests that CMR may not be reliable for evaluation of this clinical scenario [9]. However, we suspect that ICI cardiotoxicity represents a diverse and variable clinical entity, and that CMR, which is the gold standard for tissue characterization, provides valuable information to aid clinicians in making this difficult diagnosis. In our study, we systematically and comprehensively analyzed CMRs of patients referred for evaluation of suspected ICI myocarditis for quantitative strain, LGE, and T2W abnormalities.

## Materials and methods

### Study population

A clinical database search was performed to identify all patients referred for clinical CMR imaging for an indication of ICI cardiotoxicity from September 2015 to September 2019. We included all patients on ICIs with clinically diagnosed ICI cardiotoxicity based on symptoms who also had at least one of the following objective markers: either an increase in troponin, a decrease in LVEF on echocardiogram, or a new pericardial effusion. All patients referred for CMR exam met these criteria and therefore none were excluded.

Medical chart review was performed on all patients and included: demographics, cancer diagnosis, cancer treatment, cardiovascular risk factors, relevant laboratory tests (troponin, BNP, creatinine), electrocardiogram, cardiac catheterization, endomyocardial biopsy, treatment for myocarditis, other immune-related adverse events (irAEs) [17], and vital status at the time of chart review. The Yale University Human Investigation Committee approved the present analysis with waiver of informed consent.

### Cardiac imaging acquisition

All CMRs were performed in a single center on a 1.5 T clinical scanner (Aera and Avanto, Siemens, Erlangen, Germany) using standard clinical protocols. Steady state free precession (SSFP) cine imaging [repetition time (TR) = 3 ms, echo time (TE) = 1.5 ms, flip angle (FA) = 60°, 30 cardiac phases, 1.4x 1.4 x 8 mm^3^ resolution) with retrospective ECG gating was acquired in the two-chamber, three-chamber, and four-chamber views, and in contiguous short axis slices of the left ventricle. Short axis T2W black-blood fast spin echo images were used [field of view (FOV) = 360mm×270mm, TE = 65 ms, slice thickness = 8 mm, FA = 90°, bandwidth = 781 Hz/pixel, 0.8 mm resolution]. T2W mapping used T2W-prepared balanced steady-state free precession (bSSFP) single shot images acquired in 9 heart beats (TEs = 0 ms, 25 ms, 45 ms) with FOV = 360 mm x 270 mm, slice thickness = 8 mm, FA = 30°, parallel imaging = generalized autocalibrating partially parallel acquisition (GRAPPA 2), bandwidth = 1395Hz/pixel). The T2W maps were automatically generated using an exponential fit (image resolution 1.5mm×1.5mm x 8mm). LGE evaluation was performed 8–10 min after administration of 0.2 mmol/kg gadolinium contrast agent. After performing an inversion scout sequence, LGE imaging was obtained using inversion recovery gradient echo (TR/TE/θ = 6 ms/3.16ms/25°, 1.64 x 1.64 x 8 mm).

### Volumetric and functional assessment

All CMRs were independently re-processed in a uniform manner by the authors (A.Y.H, L.A.B.) using Circle Cardiovascular Imaging Inc. CMR^42^ (version 5.10.1). Analysis for volumes and function was performed using semiquantitative threshold detection technique, with manual adjustments. Trabeculations and papillary muscles were included in the left ventricular (LV) mass.

### Strain analysis

**Fig 1:**
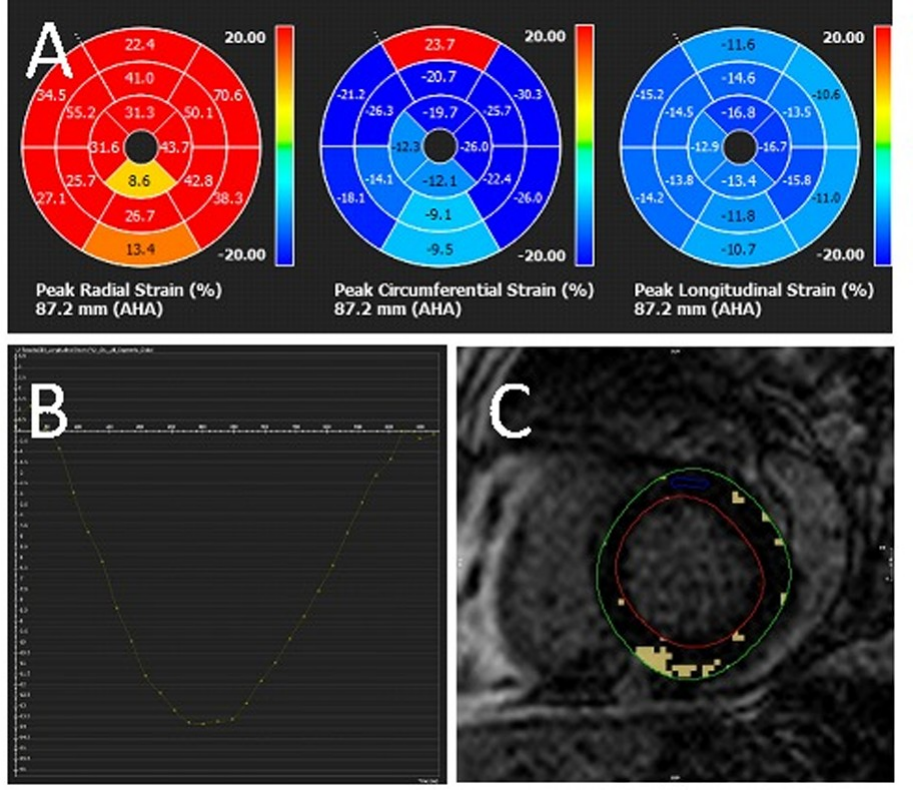
Sample CMR strain analysis. CMR showed normal LVEF (53%) with abnormal strain: A. peak global radial strain 36.4%, circumferential strain -19.7% and longitudinal strain −13.8%, B. graphical representation of peak global longitudinal strain and C. quantitative LGE of 7%.

Left ventricular GLS, global radial strain (GRS), and global circumferential strain (GCS) analysis was performed using SSFP cine short axis stack, four chamber, three chamber, and two-chamber views using CMR42 feature tracking. This software tracks image feature displacement over successive frames, similar to speckle tracking used in echocardiography [18, 19]. Representative strain analysis is shown in Fig 1. Left atrial strain was performed using CardioTrack software (Sorbonne University of Paris), a feature tracking analysis of cine SSFP acquisitions [20]. Left atrial longitudinal strain was measured and classified according to the three phases of left atrial function (reservoir (R), conduit (C) and atrial contraction (A)) phases.

### Inflammation and fibrosis assessment

**Fig 2:**
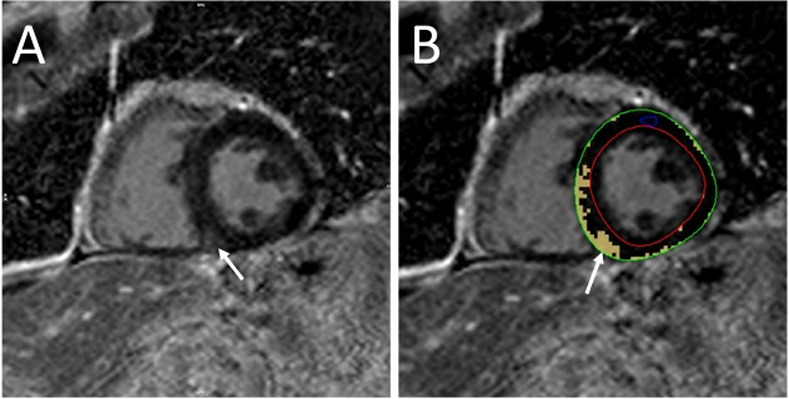
Sample quantitative late gadolinium enhancement. Mid-myocardial LGE of the basal-to-mid inferoseptal wall and inferior right ventricular insertion site (A) and quantitative scar 8.4% (B).

Myocardial edema was assessed by measuring T2 myocardium to skeletal muscle ratio (T2 ratio) [7]. Myocardial signal intensity (SI) was measured in the septum and lateral wall and skeletal muscle SI was measured in the same image. T2 ratio was calculated as: T2 ratio = SI_T2 myocardium_/SI_T2 skeletal muscle_. A ratio of ≥2 in the septum or lateral wall was considered positive for edema^8^. For T2W mapping, a T2>52ms was considered abnormal [21]. LGE was evaluated visually as present or absent and was described as ischemic or non-ischemic, based on pattern of distribution. Semi-automated quantitative LGE analysis was also performed on two-dimensional short axis LGE imaging using a cut-off of six standard deviations above normal reference myocardium. Quantitative LGE was reported as a percentage of the total LV myocardial mass. Representative quantitative LGE analysis is shown in Fig 2.

### Lake Louise Criteria

Patients were categorized according to the 2018 Updated Lake Louise Criteria for myocardial inflammation [8] as definitely positive if they had both: 1) regional increased T2W signal intensity, global increase in T2W SI ratio ≥2 or regional or global increase myocardial T2W values, and 2) abnormal LGE imaging with an area of increased SI in a nonischemic distribution.

### Statistical analysis

Descriptive statistics were expressed as median and interquartile range or number and percentage. Spearman’s rank correlation coefficient was used to describe the relationship between continuous variables and Mann-Whitney U-test was used for ordinal variables. A p-value of <0.05 was considered significant. Statistical analysis was performed using SPSS Version 26.

## Results

### Baseline characteristics and initial clinical evaluation

Twenty patients were included in the analysis. Baseline characteristics and initial clinical evaluation are reported in Table 1. The median age was 61 years old (interquartile range (IQR) 55–68 years), and 25% were female. The most common cancer diagnosis was lung (n = 6, 30%) followed by renal cell (n = 5, 25%). The majority of patients (n = 18) had metastatic disease, one patient had T1bN2 gastroesophageal cancer, and one patient had high risk myelodysplastic syndrome. Most patients were treated with nivolumab (n = 10, 50%), followed by pembrolizumab (n = 8, 40%) and ipilimumab (n = 6, 30%). Six patients (30%) received combination ICI therapy, five of which were combination nivolumab/ipilimumab. Cardiovascular risk factors were common, with 75% (n = 15) of patients with a history of smoking and 40% (n = 8) with a history of hypertension. However, only 15% (n = 3) had a history of coronary artery disease, 10% (n = 2) with prior percutaneous coronary intervention and none with a history of coronary artery bypass graft.

**Table 1:** Baseline patient characteristics.

	n %
**All**	20
**Age, mean (SD)**	61 (9.6)
**Women**	5 (25)
**Cancer diagnosis**	
Lung cancer	6 (30)
Renal cell carcinoma	5 (25)
Melanoma	3 (15)
Other	6 (30)
**Prior Therapy**	
Anthracycline	1 (5)
Monoclonal antibody (trastuzumab, bevacizumab)	4 (20)
Alkylating agent	4 (20)
Protein kinase inhibitor	3 (15)
Taxol	5 (25)
5-flurouracil	1 (5)
Carboplatin	4 (20)
Other	8 (40)
Immune checkpoint inhibitor	3 (15)
Thoracic Radiotherapy	4 (20)
**Checkpoint inhibitor at time of presentation**	
Atezolizumab	1 (6)
Pembrolizumab	6 (35)
Ipilimumab	6 (35)
Nivolumab	9 (53)
Durvalumab	2 (12)
Tremelimumab	2 (12)
Dual immune checkpoint inhibitors	6 (30)
**Past Medical History and Current Medications**	
Tobacco use	11 (55)
Hypertension	8 (40)
Myocardial infarction	2 (10)
Arrhythmia	3 (15)
Diabetes	6 (30)
CVA (TIA or stroke)	2 (10)
Hyperlipidemia	5 (25)
Coronary artery disease	3 (15)
PCI (percutaneous coronary intervention)	2 (10)
Beta Blocker	7 (35)
ACE-inhibitor/Angiotensin Receptor Blocker	6 (30)
Calcium Channel Blocker	5 (25)
Statin	7 (35)
Aspirin	6 (30)
Direct Oral Anticoagulant	4 (20)
Insulin	4 (20)
Oral diabetes medication	2 (10)
**Labs**	
Peak troponin T ng/mL, mean (SD) (reference range < .01 ng/mL)	1.2 (3.2)
B-type natriuretic peptide pg/mL, mean (SD) (reference range < 450 pg/mL)	5751 (9177)
Creatinine mg/dL, mean (SD)	1.2 (0.9)
**Concomitant immune checkpoint inhibitor related adverse events**	
Pneumonitis	3 (15)
Thyroiditis	3 (15)
Myositis	2 (10)
Colitis	1 (5)
Diabetes	1 (5)

**Fig 3:**
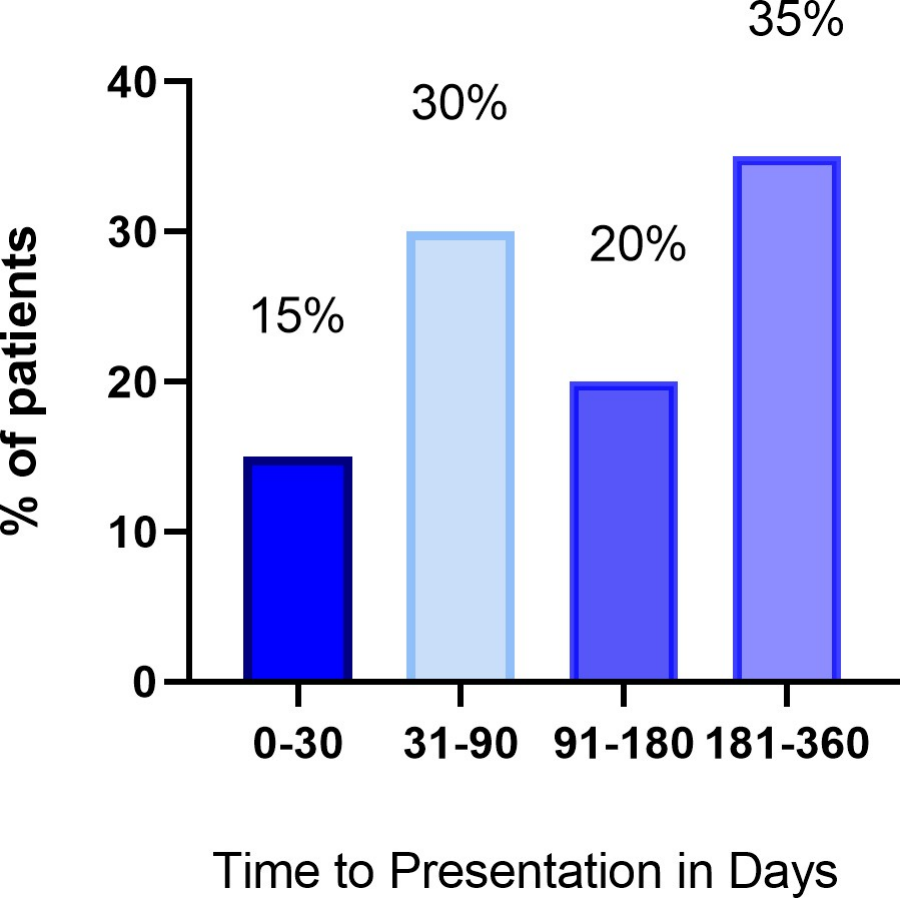
Time from starting Immune Checkpoint Inhibitor (ICI) to clinical presentation.

**Fig 4:**
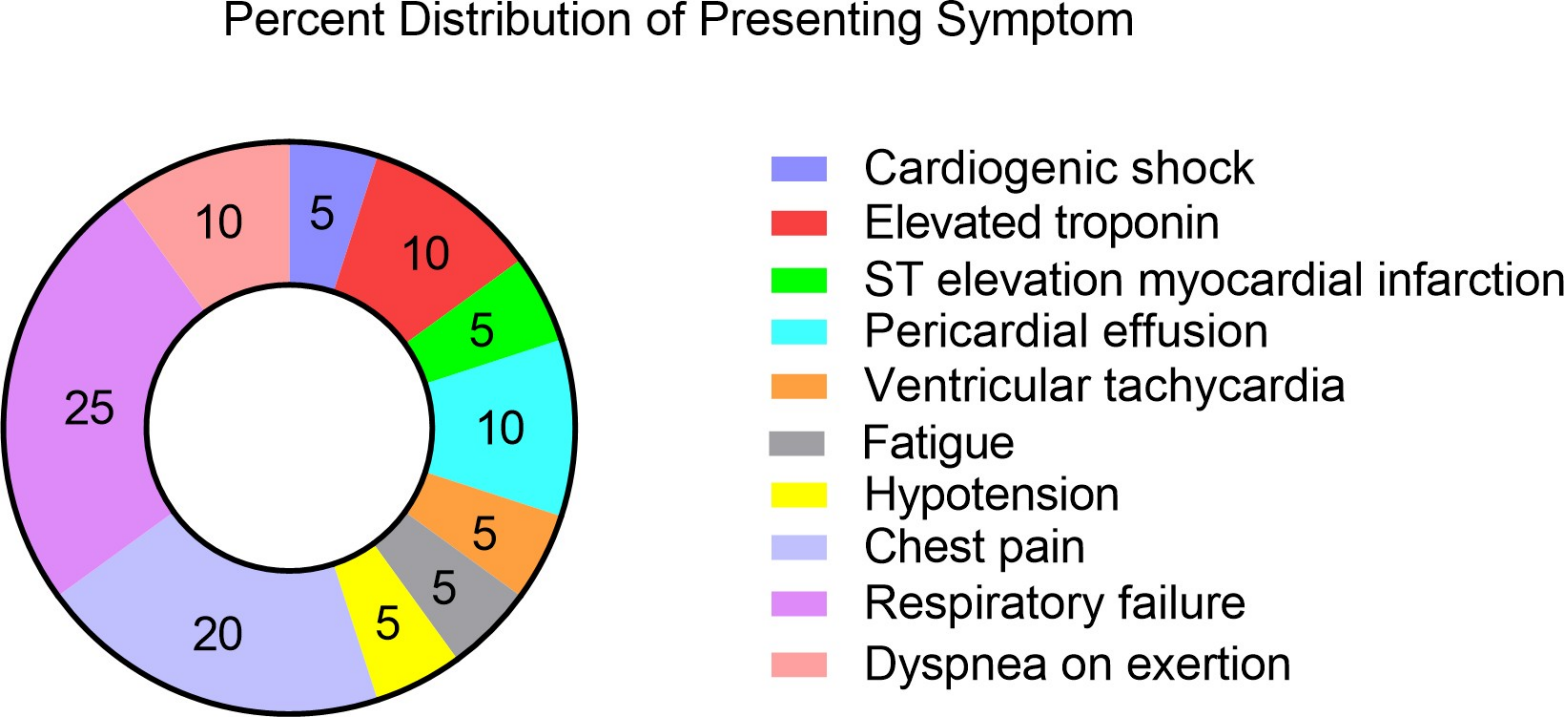
Distribution of primary symptom on presentation (n = 20).

The median time from starting checkpoint ICI therapy until presentation was 94 days (IQR 43–228 days) (Fig 3). A presenting respiratory symptom was common (n = 6, 30%), including dyspnea and respiratory failure (Fig 4). Chest pain was less common (n = 4, 20%). Troponin-T was elevated in 75% (n = 15) of patients and BNP was elevated in 12 patients (60%). The initial presenting rhythm was sinus in most patients (n = 14, 70%); however, patients also presented with ventricular tachycardia (n = 1), atrial fibrillation (n = 2), and supraventricular tachycardia (n = 3). Three patients presented with nonspecific ST or T wave changes on ECG, and one patient presented with Q waves. Nine patients (45%) had another concomitant checkpoint irAE at the time of presentation. Of these, pneumonitis (n = 3) and thyroiditis (n = 3) were the most common (Table 1).

### Functional analysis

Functional analysis is summarized in S1 Table. The median LVEF was 53% (IQR 39–60%) and half of cases (n = 10) had a normal LVEF (≥53%). The median right ventricular ejection fraction (RVEF) was 48% (IQR 45–55%).

### Myocardial strain

Feature tracking analysis revealed a median LV GLS of −11.1% (IQR −7.2− −12.6%), LV GCS of −14.4% (IQR −10.7 − −15.5%) and LV GRS of 23.0% (IQR 14.6–29.2%). There was a moderate association between GLS and left ventricular end systolic volume index (LVESVI), LVEF, and cardiac index. There was a strong correlation between LV GCS and LVESVI, LVEF, and RVEF and a moderate correlation between LV GCS and cardiac index, right ventricular end systolic volume index (RVESVI), and RV stroke volume index (RVSVI). There was a strong correlation between LV GRS and LVESVI and LVEF (r_s_ = −0.85, p <0.001). There was a moderate correlation between LV GRS and cardiac index, RVESVI, RVSVI, and RVEF.

Left atrial longitudinal strain analysis was possible for 13 patients (GLS(R) n = 13, GLS(E) n = 10, GLS(A) n = 10). The median left atrial GLS(R) was 24.5% (IQR 20.8–28.0%), GLS(E) was 11.2% (IQR 9.9–12.4%) and GLS(A) was 13.5% (IQR 10.1–14.4%). Left atrial GLS(R) was correlated with LVEF (r_s_ = 0.69, p 0.008), LV GLS (r_s_ = −0.76, p 0.003), LV GRS (r_s_ = −0.65, p 0.02), and LV GRS (r_s_ = 0.65, p = 0.02). Left atrial GLS(R) was not correlated with left atrial size (r_s_ = −0.28, p = 0.35).

### Edema imaging

T2 ratios were calculated in 17 patients and were elevated in 59% (n = 10). T2 ratio did not correlate with any CMR parameter and notably did not correlate with LVEF (r_s_ = −0.29, p = 0.25) (S2 Table). Eight of the patients with abnormal T2 ratios had qualitatively normal appearing T2W imaging. Three patients had T2W mapping, and in these patients two were abnormal. Overall, 60% of patients had abnormal T2W imaging.

### Delayed enhancement

Sixteen patients (80%) had LGE (14 non-ischemic pattern and 2 ischemic pattern). In patients with LGE, the mean percent LGE was 9.3%. Mid-myocardial right ventricular insertion site LGE was common, and occurred in 75% of patients (n = 15), and three patients had isolated RV insertion site LGE. Percent LGE did not correlate with any other CMR parameters (strain, T2W enhancement, volumes or EF) and notably did not correlate with LVEF (r_s_ = −0.29, p = 0.22) or GLS (r_s_ = 0.10, p = 0.67) (S2 Table).

### Patients with preserved left ventricular function

A LVEF ≥53% was considered normal [11] and half of the patients (N = 10) had a normal LVEF. Four of these patients had abnormal T2 ratios, all of whom had qualitatively normal T2W imaging. Six of the ten patients had LGE, and the median amount of LGE was 7.8% (IQR 3.3–10.5%). All except for one was in a non-ischemic distribution. The median GLS was −12.1% (IQR −10.8− −12.9%), GCS was −18.3% (IQR −16.6− −20.4%), and GRS was 32.4% (IQR 25.2–37.8%). In patients with a preserved LVEF compared to those with reduced LVEF, LV GLS (U = 80.50, p<0.001) and LV GRS (U = 6.00, p<0.001) differed significantly. There was no difference in age (U = 29, p = 0.12) or presence of abnormal T2W imaging (U = 47, p = 0.27), but % LGE showed a trend towards increase in subjects with reduced LVEF (U = 73, p = 0.09).

### Diagnosis and outcomes

**Fig 5:**
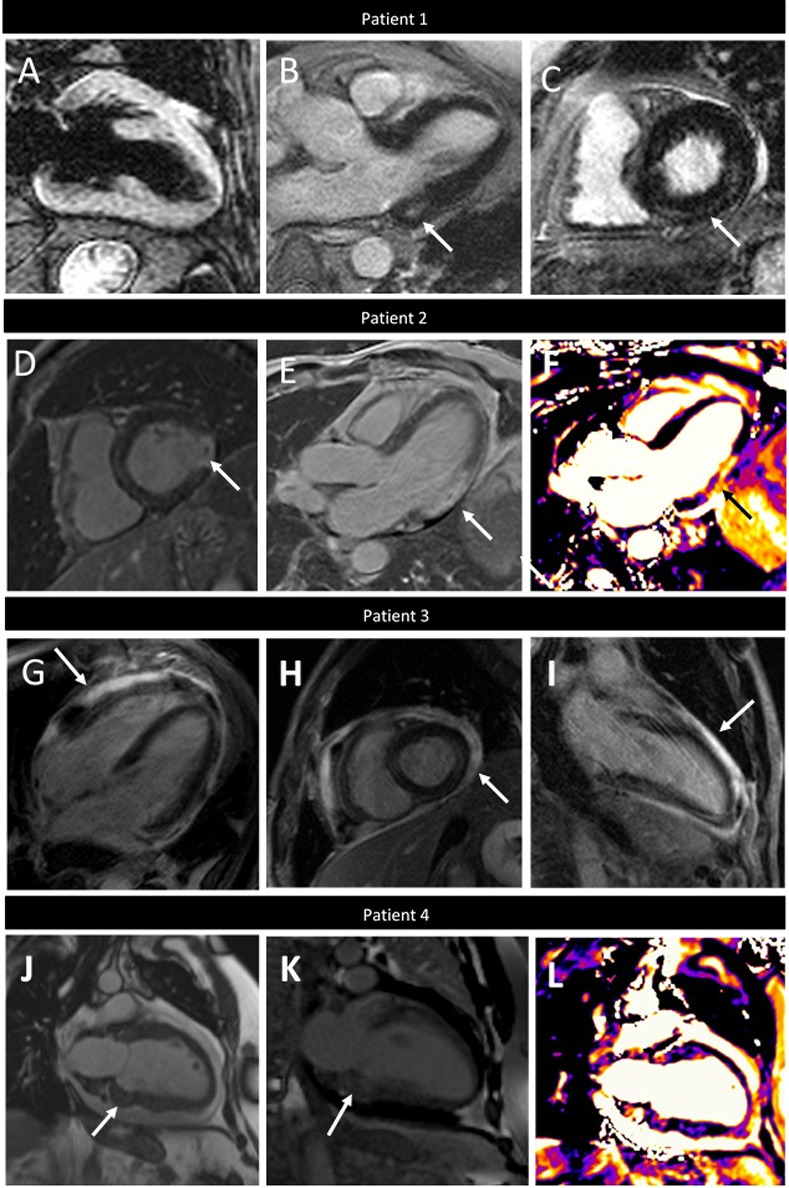
Varying CMR presentations of Immune Checkpoint Inhibitor (ICI) associated cardiotoxicity. Patient 1. Myocarditis with elevated T2-weighted signal (T2 ratio 2.2) (A) three chamber (B, white arrow) and short axis (C, white arrow) LGE imaging showing mid-myocardial LGE of basal inferior and inferoseptal walls. Patient 2. Acute myocardial infarction showing transmural LGE of the inferolateral wall with microvascular obstruction (D and E, white arrows) and corresponding increased signal on T2W-mapping (F, black arrow). Patient 3. Myopericarditis showing pericardial thickening and LGE on four chamber (G), short axis (H) and two chamber (I). Patient 4. Metastatic tumors within the basal inferior myocardium on two-chamber cine imaging (J, white arrow) with corresponding LGE (I, arrow) and edema on T2W imaging (J, black arrow).

Using the Updated Lake Louise Criteria, 9 patients (45%) were positive for definite myocardial inflammation based on having concomitant abnormal T2W imaging and LGE in a non-ischemic distribution. Representative CMR images of a patient with checkpoint inhibitor myocarditis are displayed in Fig 5 (Patient 1). Of patients who did not meet definite criteria, an additional 8 (40%) met at least one criterion (3 patients had only abnormal T2W imaging and 5 patients had only abnormal LGE). In addition to these 17 patients who met one or more criteria for myocarditis, two patients were diagnosed with myocardial infarction, based on pattern of LGE (Fig 5, Patient 2) and only one patient did not meet any criteria.

Histology was available on 7 patients, and of these, 2 patients had confirmed myocarditis (both on autopsy), 3 had inconclusive pathology (on biopsy), and 2 patients had negative pathology (on biopsy) for myocarditis. Six patients were diagnosed with concomitant pericarditis. Representative images of one patient with myopericarditis are displayed in Fig 5 (Patient 3). One patient was diagnosed with infiltrating tumors of the myocardium associated with inflammation (Fig 5, Patient 4).

Fourteen patients were treated for ICI myocarditis, and the most common treatment was steroids (100%). Other additional treatments included: infliximab (n = 3), IVIG (n = 1), mycophenolate mofetil (n = 1), tacrolimus (n = 1) and thymoglobulin (n = 1). Patients were categorized based on their clinical presentation into the following categories: treated clinical myocarditis/pericarditis (n = 14); subclinical, non-treated (n = 1); clinical, not treated (n = 3); and ischemic (n = 2). The decision to not treat those diagnosed with myocarditis was made by the treating physicians and patients in the context of their goals of care and as part of an overall risk versus benefit discussion. Of patients treated for checkpoint inhibitor myocarditis or pericarditis, 50% (n = 7) had improvement in LVEF with treatment. Of the 6 patients not treated for myocarditis, 2 had a normal LVEF at time of presentation, 3 had improvement in LVEF from abnormal to normal, and 1 did not have repeat imaging. There was no correlation between LVEF, GLS, GRS, GCS, LV volumes or % LGE and recovery of LVEF with treatment. Further, there was no correlation with presence of increased T2W signal, atrial strain, or atrial volumes and recovery of LVEF. The median follow-up time was 24 months (IQR 17–29 months), and vital status was definitively available on 17 patients. Of those, eleven patients were confirmed deceased, and of these, 4 died of cardiac causes, 6 died of cancer progression, and 1 died of multi-organ system failure due to influenza. Of the four patients who died of cardiac causes, three were treated for ICI cardiotoxicity.

## Discussion

To our knowledge, our study is the first to evaluate CMR strain, quantitative LGE, and T2-ratios in patients with ICI-associated cardiotoxicity. In this study of twenty patients with comprehensive CMR evaluation for ICI cardiotoxicity, abnormalities by CMR were detected in both patients with a depressed and with a normal LVEF, highlighting the importance of CMR for diagnosing cardiotoxicity. Specifically, in patients with a normal LVEF (≥53%), GLS was reduced at −12.1% (IQR −10.8− −12.9%) and tissue characterization (T2-ratios and quantitative LGE) was frequently abnormal.

This study also demonstrated that clinical presentation, including time to presentation and presenting symptom, is variable, as has been shown in other studies [22]. The diagnosis of ICI associated cardiotoxicity can be challenging due to the variation in clinical presentation and heterogeneous disease process. However, making a diagnosis is critical due to the high fatality rate, which needs to be weighed against the consequences of administering versus withholding aggressive immune suppression in patients on potentially life-saving therapy. Similar to Zhang et al [9] and in accordance with published criteria [23], we used a combination of clinical and objective evidence of cardiotoxicity to define our cohort. While some patients in our study presented with overt cardiotoxicity in the form of ventricular tachycardia or cardiogenic shock, others presented with elevation in troponin or dyspnea on exertion. Many presented with respiratory failure, which can be multifactorial in patients with cancer, especially those with lung cancer or with lung metastases. This variable clinical presentation is consistent with previous studies [24]. Troponin was elevated in 75% of patients; however, four patients with clinically diagnosed and treated ICI myocarditis did not have elevated troponin. Our findings confirm previous studies that have suggested a high index of clinical suspicion is needed in patients on ICI and that ICI cardiotoxicity is a heterogenous disease process.

We found that strain parameters correlated well with LV function; however, even in patients with normal LVEF, strain imaging was abnormal. There is some variability, due to method and software used, in normal values for CMR strain using FT [25]. However, in a recent meta-analysis of 659 healthy subjects, the mean LV GLS was −20.1% (95% CI: −20.9% to −19.3%) [26]. Similarly, in a study of 40 healthy controls the average GLS was −19.3 ± 1.9% by Circle CVI FT [27]. In echocardiography a GLS value of −19% is used as a cut off for normal in evaluation of chemotherapy-induced cardiotoxicity [11]. In our study, the median LV GLS of patients with an LVEF ≥53% was −12.1% (IQR −10.8− −12.9%), much lower than reported normal values, signaling abnormal tissue function despite normal LVEF. Recent studies have shown abnormal CMR strain in patients with suspected non-ICI associated myocarditis and normal LVEF [13], as well as abnormal strain by echocardiography in patients with ICI myocarditis and normal LVEF [15]. Our findings are in accordance with this recently published data; however, we have shown, for the first time, abnormal FT strain by CMR in patients with ICI cardiotoxicity, both with an abnormal and a normal LVEF. Abnormal left atrial strain by CMR has been described in acute myocarditis and has been shown to have incremental value in the diagnosis of myocarditis compared with Lake Louise Criteria alone [16]. We found left atrial GLS (R), GLS (E) and GLS (A) values that were similar to previously published normal values, although only 13 of our subjects had images available for LA strain interpretation [28]. Left ventricular strain imaging by echocardiography is well accepted in chemotherapy-induced cardiomyopathy as an early sign of cardiac dysfunction [10]; however, this is the first study evaluating CMR strain imaging in ICI cardiotoxicity. Advanced analysis with CMR strain may help identify patients with more subtle or early ICI cardiotoxicity. Further studies comparing CMR strain patterns in patients on ICI with and without suspected ICI cardiotoxicity would be beneficial.

Late gadolinium enhancement and abnormal T2W imaging are established criteria for the diagnosis of myocarditis by CMR [7, 8]. Further, LGE is an independent predictor of mortality in acute myocarditis [29]. Abnormal LGE was common in our cohort of patients (75%, n = 15) and most were in a non-ischemic distribution. Quantitative LGE did not correlate with any CMR parameters and did not correlate with LVEF (r_s_ = −0.29, p = 0.22) or GLS (r_s_ = 0.10, p = 0.67), but showed a modest trend in correlation with troponin (r_s_ = 0.43, p = 0.07). This is contrary to a previous study which showed that quantitative LGE had a significant, albeit weak, inverse correlation with LVEF in acute myocarditis [30]. It is possible that our differing results are due to sample size limitations; alternatively, ICI myocarditis may be distinctly different than other forms of myocarditis in CMR findings. In a recent study of 103 patients with ICI associated myocarditis, LGE was reported in only 48% of cases; however, quantitative evaluation was not performed [9]. This study also found that qualitative T2W imaging was abnormal in only 28% of patients. While qualitatively abnormal T2W imaging was rare in our study as well, we found that quantitative T2W values were elevated in 60%, suggesting an increased sensitivity of this approach. In our study, T2 ratios did not correlate with any CMR parameters, including LVEF (r_s_ = −0.29, p = 0.25). These findings are important, as LVEF, particularly by echocardiogram, is used as a screening tool for diagnosis of myocarditis. LVEF alone may miss more subtle tissue abnormalities that precede overt cardiac dysfunction. Overall, almost half of the patients in our study met full criteria for acute myocarditis based on the Updated Lake Louise Criteria, and 85% met one main criterion, which is supportive of the diagnosis of acute myocarditis in the correct clinical context [8].

Regarding the pattern of LGE, we found that most patients had a non-ischemic distribution, but two patients were diagnosed with ischemic disease based on pattern of LGE. One of these patients had diffuse increase in T2 ratio outside of the area of the ischemic pattern LGE, suggestive of myocarditis and prior infarct. The second patient clearly had acute infarct as with concomitant increased T2 signal mostly within the area of LGE and subtle patchy increased signal outside of the area of infarct. Neither of these patients were given a diagnosis of definite myocarditis by CMR in our study, due to the presence of ischemic LGE. Acute coronary plaque rupture [31] and non-coronary acute arterial thromboembolic events [32] have been described as a potential consequence of ICI therapy and it is known that T-cells and immune checkpoint proteins play a role in formation and stabilization of atherosclerotic plaque [33]. Whether the acute coronary syndrome was coincidental or secondary to ICI therapy is unknown; however, given the role of checkpoint proteins in plaque stabilization, we suspect it is related. Additionally, recent study showed that use of ICIs is associated with an increase in myocardial infarction rate as well as atherosclerotic plaque progression by computed tomography [34]. In our study, of the 15 patients with elevated troponin, 7 had a left heart catheterization (LHC) to exclude ischemia. Of the 13 patients who did not have a LHC, 11 had at least one T1 or T2 abnormality on CMR suggestive of myocarditis.

We also found that RV insertion site LGE was common. Right ventricular insertion site LGE can be seen in other non-ischemic cardiomyopathies (NICM), and is frequently seen in patients with hypertrophic cardiomyopathy [35], and an analysis of patients with other NICM found that patients with this pattern did not have significantly increased adverse outcomes [36]. Right ventricular insertion site LGE with histopathological evidence of inflammation and fibrosis has been described in a case report of a patient with ICI myocarditis from our institution [37]. However, the significance of RV insertion site LGE in patients with myocarditis is not known.

One patient with myocardial tumor infiltration from metastatic disease did demonstrate evidence of myocardial inflammation by T2W mapping, positive LGE, and positive troponins (Fig 5, Patient 4). Without having a baseline CMR and troponin, it is not known if the myocardial inflammation was from the tumor infiltration itself or secondary to the ICI therapy, either as a cardiotoxic complication or just as a complication of the therapy effectively attacking the tumor cells. Regardless, this patient did present with heart failure symptoms and ultimately died from his cardiac disease.

CMR is a well-established tool for the diagnosis of non-ICI related myocarditis [7, 8], as discussed above. Interestingly, we found that quantitative abnormalities in LV strain, LGE, and T2W imaging were very common, even in patients with preserved LVEF. Tissue characterization, particularly in patients with preserved LVEF, can help identify abnormalities indicative of an underlying myopathic process, highlighting the utility of CMR in making this challenging diagnosis.

### Study limitations

This study is limited by its retrospective nature and small sample size from a single institution. This is, however, the most comprehensive quantitative evaluation of CMRs in this population and the first study to evaluate strain by CMR and quantitative LGE. Second, as these are clinically performed CMR exams, we do not have uniform CMR data acquired on all patients. Many patients did not have suitable images for left atrial strain, although almost all had suitable images for left ventricular strain and none had limitations for analysis of all other LV parameters. Only one patient had a limitation in images for LV strain analysis. Exclusion of cases with suboptimal quality for left atrial strain analysis may introduce bias and should be interpreted with caution. Also, while most patients had standard T2W imaging, most did not have T2W mapping due to variability amongst scanners. Additionally, T1 mapping was not available, which is a limitation, as extracellular volume concentration as derived from T1 mapping can be used for detection of myocarditis per the updated Lake Louise Criteria. Future studies utilizing T2W and T1W mapping may be more sensitive and specific for identification of inflammation.

Additionally, we did not have endomyocardial biopsy (EMB) on the majority of patients. This is in concordance with contemporary clinic practice, where a non-invasive assessment and diagnosis of myocarditis is most often initially pursued in lieu of EMB, as discussed by Friedrich MG specifically in the context of ICI associated myocarditis [38]. Furthermore, published CMR studies have utilized the clinical diagnosis of myocarditis for a reference standard in other myocarditis populations [39, 40]. In light of this practice, the most recently published paper by Zhang et al. only had histology in about half of patients. In general, the sensitivity of right ventricular EMB in the setting of myocarditis is not high, prone to sampling error, and has been reported as low as 45% in lymphocytic myocarditis [41, 42].

Lastly, while we cannot exclude prior chemotherapy as a contributing factor to the abnormalities in tissue characterization, all patients had no known cardiac dysfunction prior to ICI therapy. However, the lack of routine baseline cardiac function assessment and monitoring in this cohort is a limitation to our study.

## Conclusion

In this study, we performed a comprehensive CMR evaluation of patients with ICI cardiotoxicity and demonstrated the value of LV strain and tissue characterization for diagnosis. Importantly, patients with a normal LVEF demonstrated abnormal left ventricular strain, increased T2W signal, and abnormal LGE imaging. ICI cardiotoxicity requires a high level of suspicion and CMR is a sensitive tool to detect changes in myocardial tissue and function. The utilization of CMR for detection of ICI cardiotoxicity should be considered when making this challenging diagnosis in clinical practice, even in the setting of preserved LV systolic function on echocardiography. Future prospective CMR studies would be of value in this patient population and should include quantitative techniques, such as strain analysis and parametric mapping techniques, for sensitive detection of ICI cardiotoxicity.

## Supporting information

S1 Table Cardiac magnetic resonance imaging functional analysis. (DOCX)

S2 Table (PDF)
