# Supplementary material for: Left ventricular myocardial strain and tissue characterization by cardiac magnetic resonance imaging in immune checkpoint inhibitor associated cardiotoxicity
Source: PLoS One. 2021 Feb 19;16(2):e0246764. doi: 10.1371/journal.pone.0246764 (PMC7895343; doi:10.1371/journal.pone.0246764)
Supplement: S1 Table — (DOCX) [file pone.0246764.s001.docx]

**S1 Table.** Cardiac magnetic resonance imaging functional analysis.

| Parameter | Median | Interquartile Range |
| --- | --- | --- |
| Left atrial area (cm^2^) | 23.0 | 20.8-25.3 |
| Right atrial area (cm^2^) | 20.0 | 18.9-22.5 |
| Left ventricular end diastolic volume index (mL/m^2^) | 70.0 | 61.0-90.0 |
| Left ventricular end systolic volume index(mL/m^2^) | 32.0 | 25.5-43.0 |
| Left ventricular stroke volume index (mL/m^2^) | 37.5 | 28.3-44.0 |
| Left ventricular ejection fraction (%) | 52.5 | 38.3-62.3 |
| Left ventricular cardiac output (L/min) | 5.9 | 4.2-6.8 |
| Left ventricular cardiac index output (L/min/m^2^) | 3.1 | 2.3-3.5 |
| Left ventricular mass (g) | 108.0 | 99.8-143.5 |
| Left ventricular mass (g/ m^2^) | 58.5 | 54.8-65.5 |
| Right ventricular end diastolic volume index (mL/m^2^) | 81.5 | 68.8-93.0 |
| Right ventricular end systolic volume index (mL/m^2^) | 77.5 | 66-103.3 |
| Right ventricular stroke volume index (mL/m^2^) | 74 | 58.5-86.3 |
| Right ventricular ejection fraction (%) | 48 | 43.5-53.3 |
| Right ventricular cardiac output | 5.9 | 5.1-7.0 |
| Right ventricular cardiac index | 3.3 | 2.7-3.5 |
