# Supplementary material for: Left ventricular myocardial strain and tissue characterization by cardiac magnetic resonance imaging in immune checkpoint inhibitor associated cardiotoxicity
Source: PLoS One. 2021 Feb 19;16(2):e0246764. doi: 10.1371/journal.pone.0246764 (PMC7895343; doi:10.1371/journal.pone.0246764)
Supplement: S2 Table — (PDF) [file pone.0246764.s002.pdf]

**Age**<br>Age at presentati	**Gender**<br>0=male, 1=female	**Cancer diagnosis**<br>0= lung cancer, 1=renal cell,	LVEF	LVEDV	LVEDVI	LVESV	LVESVI
45	1	2	13	157	93	136	81
64	1	0	54	140	70	64	32
73	0	4	52	128	71	61	34
78	1	5	62	120	70	46	26
53	0	2	16	307	161	258	135
61	0	0	68	139	64	45	21
73	0	0	40	117	60	71	36
51	0	1	60	122	61	59	24
52	0	0	21	85	43	68	34
67	0	0	53	147	68	70	32
74	0	0	66	134	61	48	22
55	1	3	20	163	90	130	72
66	0	5	33	204	121	121	72
56	0	5	63	128	73	47	27
56	0	1	52	196	95	95	42
60	0	1	65	171	84	59	29
60	0	1	49	168	90	86	46
45	1	2	51	108	64	53	31
65	0	1	82	95	51	18	9
70	0	5	57	95	51	40	22

LV-SV	LV-SV-I	CO	CI	HR	LV mass	LV mass index	RVEF	RVEDV
21	12	2.5	1.5	120	102	61	14	144
76	38	5.6	2.8	72	120	60	42	177
67	37	6.4	3.6	100	100	56	48	130
75	43	6.7	3.8	91	111	64	44	158
50	26	3.6	1.9	64	157	82	17	283
94	44	9.4	4.3	94	145	67	54	167
46	24	3.7	2	81	106	54	54	117
74	37	6.8	3.4	88	99	50	48	154
18	8.8	3	1.5	173	96	52	33	94
77	36	6.8	3.2	85	110	51	52	165
86	39	9.1	4	97	143	65	53	143
33	18	3	1.7	92	103	57	23	191
53	49	5.6	3.3	71	149	88	51	191
81	46	8.6	4.9	100	98	56	55	157
101	45	7.3	3.3	74	166	74	47	225
112	55	6.1	3	55	123	60	64	186
82	44	5.5	2.9	65	159	88	44	184
56	33	4.3	2.5	76	93	55	51	106
77	41	6.6	3.5	65	84	45	74	107
55	29	4.5	2.4	85	103	55	46	130

RVEDVI	RVESV	RVESVI	RV-SV	RV-SV-I	R-CO	R-CI	[illegible] (0= non ischemic; 1=ischemic)
86	125	74	19	12	2.3	1.4	0
88	103	51	74	37	5.5	2.7	0
73	67	37	63	35	6	3.4	0
91	89	91	69	40	6.2	3.5	0
148	235	123	48	25	3.5	1.9	0
77	76	35	90	42	9.0	4.2	0
60	54	28	63	32	5.1	3	1
77	81	40	74	37	6.5	3.3	0
47	63	32	31	15	5.2	2.6	0
77	79	37	86	40	7.5	3.5	0
65	61	28	82	38	8.5	3.9	0
106	147	81	44	24	4	2.2	0
114	95	56	96	57	5.8	3.6	0
89	70	40	87	49	9.2	5.3	0
100	120	53	105	47	7.6	3.4	1
91	67	33	119	58	6.5	3.2	0
99	104	56	80	43	5.3	2.9	0
62	52	30	54	32	4.1	2.4	0
57	27	15	80	42	6.8	3.6	0
70	70	37	60	32	5	2.7	0

[illegible] site LGE (0=N; 1=Y)	**% LV SCAR**	LV-GLS	LV-GCS	LV-GRS	LA SIZE Quant cm2	RA SIZE Quant cm2	Pericardial effusion 0=no, 1=yes	pericardial LGE 0=no, 1=yes
1	15	-1.9	-1.8	1.1	28	20	0	0
0	0	-11.9	-15.5	25	28	19	0	0
1	5.7	-12.7	-15.9	22.4	23	19	0	0
0	0	-13		19	25	22	0	0
1	2.4	-2.2	-6.4	10.6	31	24	0	0
0	1.3	-10.4	-21.8	38.3	22	16	1	1
1	23.5	-6.1	-12.4	21.7	25	18	0	0
1	8.4	-12.5	-15.2	16.1	16	21	0	1
1	13.4	-3.3	-5.3	3.3	20	20	1	0
1	7.2	-13.8	-19.7	36.4	21	28	1	1
1	14.1	-9.8	-16.6	25.6	16	18	1	0
1	2.3	-6.7	-8.8	9.1	25	19	1	0
0	0	-7.3	-11.9	15.9	21	22	1	0
1	0	-12.1	-17.4	31.6	23	20	1	1
1	16.2	-12.1	-12	15.1	26	26	0	0
0	0	-9.4	-20.4	39.2	23	26	0	0
1	16.5	-8.7	-9.4	13	22	20	1	0
1	0.7	-12.7	-16.7	22.9	31	31	1	1
1	11.2	-18.2	-29	64.6	17	13	0	0
1	2	-11.8	-17.4	28.4	19	13	0	0

Left atrial GLS	Left atrial GLS	Left atrial GLS	Left atrial GLS rate	Left atrial GLS rate	Left atrial GLS rate	T2 Septum	T2 Lateral
GLSr	GLSe	GLSa	srGLSr	srGLSe	srGLSa		
NA	NA	NA	NA	NA	NA	136	198
20.8	11	9.8	0.7	-0.9	-2	137	192
26.4	11.8	14.6	1.1	-1	-1.6		
NA	NA	NA	NA	NA	NA	150	180
5.2	2.5	2.7	0.3	-0.2	-0.2	186	121
NA	NA	NA	NA	NA	NA	147	151
23.7	9.8	13.8	1	-0.9	-1.3	230	209
24.5	11.4	13.1	1.1	-1	-1.5	157	124
NA	NA	NA	NA	NA	NA	147	183
22.6	NA	NA	1.1	-1.4	NA	213	188
28	NA	NA	1.5	-2	NA	149	183
11.7	NA	NA	0.7	-0.8	NA	210	242
20	12.6	7.4	0.8	-1.1	-0.7	319	314
NA	NA	NA	NA	NA	NA	161	190
27	10	17.1	1.6	-0.7	-1.5		
32.8	21.9	10.9	1.3	-1.1	-0.6	107	137
NA	NA	NA	NA	NA	NA		
28.3	14.5	13.8	1.2	-1.3	-0.7	172	196
NA	NA	NA	NA	NA	NA	223	252
28.8	9.1	19.7	1.1	-0.9	-2.1	268	184

T2 Muscle	T2 Ratio Septum	T2 Ratio Lateral	T2 Ratio (mean)	T2 value on map	Pericardial effusion 0=no, 1=yes	pericardial LGE 0=no, 1=yes
68	2.0	2.9	2.5		0	0
80	1.7	2.4	2.1		0	0
				46	0	0
109	1.4	1.7	1.5		0	0
127	1.5	1.0	1.2		0	0
130	1.1	1.2	1.1		1	1
63	3.7	3.3	3.5		0	0
92	1.7	1.3	1.5		0	1
86	1.7	2.1	1.9		1	0
91	2.3	2.1	2.2		1	1
61	2.4	3.0	2.7		1	0
61	3.4	4.0	3.7		1	0
137	2.3	2.3	2.3		1	0
107	1.5	1.8	1.6		1	1
				91	0	0
69	1.6	2.0	1.8		0	0
				64	1	0
113	1.5	1.7	1.6		1	1
110	2.0	2.3	2.2		0	0
110	2.4	1.7	2.1		0	0
